# Patterns of relatedness and genetic diversity inferred from whole genome sequencing of archival blood fluke miracidia (*Schistosoma japonicum*)

**DOI:** 10.1371/journal.pntd.0009020

**Published:** 2021-01-06

**Authors:** Zachary L. Nikolakis, Nicole R. Hales, Blair W. Perry, Drew R. Schield, Laura E. Timm, Yang Liu, Bo Zhong, Katerina J. Kechris, Elizabeth J. Carlton, David D. Pollock, Todd A. Castoe

**Affiliations:** 1 Department of Biology, University of Texas at Arlington, Arlington, Texas, United States of America; 2 Department of Biochemistry & Molecular Genetics, University of Colorado School of Medicine, Aurora, Colorado, United States of America; 3 Institute of Parasitic Disease, Sichuan Center for Disease Control and Prevention, Chengdu, The People’s Republic of China; 4 Department of Biostatistics and Informatics, Colorado School of Public Health, University of Colorado, Anschutz, Aurora, Colorado, United States of America; 5 Department of Environmental and Occupational Health, Colorado School of Public Health, University of Colorado, Anschutz, Aurora, Colorado, United States of America; Wellcome Trust Sanger Institute, UNITED KINGDOM

## Abstract

Genomic approaches hold great promise for resolving unanswered questions about transmission patterns and responses to control efforts for schistosomiasis and other neglected tropical diseases. However, the cost of generating genomic data and the challenges associated with obtaining sufficient DNA from individual schistosome larvae (miracidia) from mammalian hosts have limited the application of genomic data for studying schistosomes and other complex macroparasites. Here, we demonstrate the feasibility of utilizing whole genome amplification and sequencing (WGS) to analyze individual archival miracidia. As an example, we sequenced whole genomes of 22 miracidia from 11 human hosts representing two villages in rural Sichuan, China, and used these data to evaluate patterns of relatedness and genetic diversity. We also down-sampled our dataset to test how lower coverage sequencing could increase the cost effectiveness of WGS while maintaining power to accurately infer relatedness. Collectively, our results illustrate that population-level WGS datasets are attainable for individual miracidia and represent a powerful tool for ultimately providing insight into overall genetic diversity, parasite relatedness, and transmission patterns for better design and evaluation of disease control efforts.

## Author summary

Schistosomiasis is a neglected tropical disease caused by helminths within the genus *Schistosoma*, which affects over 200 million people globally. Here, we demonstrate the ability to obtain whole genome sequences from single *S*. *japonicum* miracidia that have been archived for years, and the promise of how these data may be used to answer major questions to understand and eventually help control schistosomiasis. To demonstrate the approach, we generated a moderate-sized dataset of 22 individual *S*. *japonicum* genomes and use these data to infer fine-scale patterns of relatedness among parasites from human hosts from two villages in Sichuan, China. We highlight how this type of genomic information may be useful for understanding the genetic variation among individual parasites with high precision, which could ultimately provide high resolution for understanding transmission pathways and inform control efforts with larger sample sizes. We also show that it is possible to use less data per individual than we obtained here, and still maintain high levels of accuracy; this would enable greater sample sizes by lowering individual sample cost. Collectively our results demonstrate that sampling genomes of individual schistosome parasites is now feasible on a population-level scale, opening new avenues for studying relatedness, tracing parasite spread, and studying parasite biology.

## Introduction

An estimated one billion people globally are impacted by schistosomiasis and other human helminthiases [1–3] Global efforts are underway to control and, in some cases, eliminate these parasitic infections [4]. Genomic approaches can help advance these efforts, opening up new methods to evaluate treatment failure, monitor for drug resistance, and infer transmission pathways. Previous studies used small-scale genotyping approaches (e.g., microsatellite genotyping) to study parasite population structure and transmission (e.g., [5–9]), but the low number of genetic markers available for such studies limits the resolution of inferred transmission patterns. Recently, higher resolution reduced-representation genomic methods (i.e., double-digest RAD-seq [10]) have been used to infer parasite population genetic structure and transmission patterns [11]. These studies showed that degrees of parasite diversity and relatedness vary within- and between-hosts, highlighting the utility of large genetic data sets to understand fine-scale transmission pathways [12]. However, even these higher-resolution data have limited utility for inferring variation among functional genomic regions (e.g., protein-coding diversity) that may be important for understanding responses of the parasite population to control-driven selection.

In contrast, the increasing feasibility of leveraging whole genome sequencing (WGS) for the study of schistosomes provides an opportunity to estimate genetic diversity, relatedness, and population structure at a previously unattainable degree of resolution. Furthermore, WGS data can identify patterns of variation in functional regions associated with control-driven selection on parasite populations, and potentially detect genomic evidence of emerging drug resistance. To date, however, population genomic studies of schistosomiasis have been limited by the need to pool individual samples to obtain adequate DNA, resulting in estimates of broad regional patterns of parasite genetic structure [13–15]. Recently, several studies demonstrated that individual miracidia can be effectively used for large-scale genotyping through the use of whole genome amplification (WGA), thereby circumventing limitations imposed by low yield of single miracidium samples [11,12,16,17].

In this study, we demonstrate the feasibility and utility of leveraging WGA to conduct whole genome sequencing for individual archived *S*. *japonicum* miracidia collected from human hosts in Sichuan, China–an important region of re-emergence and persistence of schistosomiasis despite ongoing control efforts [18–20]. We demonstrate the application of single-miracidium-derived whole genome data to discern genetic structure and patterns of relatedness within and among hosts and villages, and discuss how similar analyses that include greater population sampling could be used to define next-step priorities for control measures. Additionally, we conduct analyses to examine the feasibility of using lower-coverage whole genome datasets to understand how lower-coverage sampling can enable increased economy of future WGS data collection. An empirical understanding of this trade-off between depth of coverage and number of individuals sequenced has important implications for the design of future population-based studies to include greater numbers of parasite samples at lower per-sample costs.

## Materials and methods

### Ethics statement

Sample collection was approved by the Sichuan Institutional Review Board, the University of California, Berkeley, Committee for the Protection of Human Subjects, and the Colorado Multiple Institutional Review Board (15–1059). All participants provided written, informed consent. Participants who tested positive for *S*. *japonicum* were notified and referred to the local anti-schistosomiasis control station for treatment.

### Sample collection

A total of 22 archived field-collected samples of *S*. *japonicum* miracidia were obtained from 11 infected humans in Sichuan Province, China in 2016 during village-wide infection surveys using methods described in [19,21] as part of an ongoing study of the reemergence and persistence of schistosomiasis in regions targeted for elimination. Individual miracidia were collected from two villages, denoted as village C and M, in which village C was sampled using RADseq data in a previous study [12]. Generally, we designed this sampling to test our approach for surveying multiple fine-scale levels of relatedness, including: within-host, between hosts from a single village, and between hosts from different nearby villages. Miracidia were collected from 10 human hosts in village C and 1 human host in village M (Tables 1 and 2). Village C is a small village (approximate population: 54 people over age 5) located approximately 15 km from a major road. Infection prevalence in village C, as assessed by the concurrent infection surveys was 27% and the hosts included here represent 10 of the 13 village residents that tested positive for *S*. *japonicum* infection. Village M is a larger village (approximate population: 212 people over age 5) located next to a major road. The infection prevalence in village M was 17% with 24 village residents testing positive, 1 of whom is included here. These villages are separated by a Euclidean distance of 12 kilometers, or 17 kilometers by road (S1 Fig). Briefly, participants were tested for infection using the miracidia hatching test and miracidia were collected from positive hatching tests. Miracidia were collected from the top of the hatching test flask, isolated using a hematocrit tube or Pasteur pipette drawn to a narrow bore with a flame, washed three times with autoclaved deionized water, and placed on a Whatman FTA indicating card (GE) for long-term storage. After drying, cards were stored in a desiccator at room temperature.

**Table 1:** Characteristics of the individuals from whom miracidia were collected.

Host	Village	Household	Sex	Age*	Occupation	Infection intensity (EPG)+	Miracidia¶
1	C	1	Male	40–49	Farmer		17090A, 17090B
2	C	1	Female	40–49	Farmer		17082B, 17082C
3	C	2	Female	80–89	Homemaker	120	17048B, 17048C
4	C	3	Male	70–79	Farmer	96	17103A, 17103B
5	C	4	Female	60–69	Farmer	8	17088A, 17088B
6	C	5	Female	50–59	Homemaker	0	17064A
7	C	6	Male	50–59	Farmer	80	17112A
8	C	7	Female	40–49	Farmer		17059D
9	C	8	Male	80–89	Farmer	48	17052A, 17052B
10	C	9	Female	50–59	Farmer		17053B
11	M	10	Female	70–79	Farmer		17128B, 17128C, 17131C, 17141B, 17148B, 17148C

*To protect the privacy of participants, age is provided in 10 year age bands.

+Infection intensity was measured using the Kato-Katz method, examining three slides for each participant. Participant 5 tested positive by hatch tests but had no detectable eggs. Infection intensity measurements are missing for four participants.

¶In red are the two miracidia with low frequency of mapped reads that were subsequently excluded from downstream analyses.

**Table 2:** Mapping and coverage statistics for each WGS sample. Total reads, proportion of mapped reads and central tendency statistics on exon coverage reported.

Miracidium ID	Village alt ID	Host ID	Total Reads (Q>20)	Total Mapped Reads	% Mapped Reads	Mean Genomic Coverage (50Kb)	Median Genomic Coverage (50Kb)	Mean Exon Coverage	Median Exon Coverage	Mode Exon Coverage
17090B	C	1	242964690	224059064	92.22	463	25	237.7698	45	18
17090A	C	1	121503915	112818813	92.85	180	16	103.3254	28	12
17082C	C	2	317925609	299109366	94.08	699	25	354.9228	49	27
17082B	C	2	203315847	183415003	90.21	242	22	258.8655	45	21
17048B	C	3	256689097	230890466	89.95	180	33	342.9313	75	39
17048C	C	3	202070081	183539245	90.83	247	22	176.3338	43	21
17103B	C	4	830095814	773700617	93.21	1507	80	901.8963	174	107
17103A	C	4	128019168	120061639	93.78	120	21	107.8554	36	14
17088A	C	5	173672554	161720975	93.12	196	19	248.9284	44	29
17088B	C	5	52399022	48480107	92.52	88	7	68.3051	12	6
17064A	C	6	194083616	185010305	95.33	657	7	101.0228	13	6
17112A	C	7	227823406	218704118	96	952	9	82.84688	15	5
17059D	C	8	513724448	493482501	96.06	1512	27	632.2784	57	21
17052A	C	9	271323109	58201626	21.45	77	1	76.39758	0	0
17052B	C	9	234545104	223500519	95.29	697	19	286.8104	34	11
17053B	C	10	234200156	214819846	91.72	153	42	256.398	93	62
17148C	M	11	289746165	273320135	94.33	210	51	372.1544	116	64
17141B	M	11	230826536	215001306	93.14	194	38	340.5262	81	46
17148B	M	11	410543401	338471019	82.44	338	34	277.2063	80	34
17128C	M	11	289249117	271422649	93.84	984	18	173.9289	34	17
17131C	M	11	108726702	99523184	91.54	100	16	101.7835	31	14
17128B	M	11	463499246	95771466	20.66	165	1	34.76015	0	0

### Whole genome amplification and sequencing

Due to the limited amount of DNA available from a single miracidium (~2ng), whole genome amplification (WGA) was used, similar to previous studies that have used this approach to sequence reduced representation libraries [11]. Individual miracidia were extracted from Whatman cards using a Whatman Harris 2mm micro-core punch (Whatman; cat. WB100029). Following excision, punches underwent five consecutive 5-minute washes, three washes with FTA buffer and two washes with 200uL TE buffer. After the final wash, punches were left to dry for 30min-60min at room temperature. DNA from each miracidium was amplified directly from the punch using the Illustra Ready-To-Go GenomiPhi V3 DNA Amplification Kit (GE Healthcare; cat. 25-6601-96) following the manufacturer’s recommended protocol for amplification with minor adjustments made to accommodate amplification from a 2mm disk. Specifically, dried disks were transferred to an amplification tube containing 20uL of 1x denaturation buffer. Tubes were incubated for 95°C for 3 minutes and then immediately placed on ice. Liquid from the tube was then added to an individual amplification pellet provided in the kit. The pellet dissolved over the course of 10min, incubated on ice. After gentle mixing, the liquid was transferred back to its original tube with the 2mm disk. To amplify, each reaction was then subjected to 90 minutes of amplification at 30°C, followed by enzymatic heat kill at 65°C for 10 minutes, and held at 4°C until they were moved to a -20°C freezer for storage. Following WGA, DNA was quantified using a Qubit dsDNA Assay Kit (Invitrogen), and only samples containing >200ng were used in downstream shotgun genome library preparation. For each sample, 200ng of WGA DNA was used to construct libraries using the KAPA HyperPlus kit (KAPA Biosystems; cat. KK8514). Each sample was prepared using half-reactions and uniquely indexed using IDT for Illumina TruSeq UD Indexes (Illumina; cat. 20022370). All 22 individual libraries were multiplexed into a single, pooled library and sequenced on a single Illumina NovaSeq lane using 150bp paired-end sequencing.

### Sequencing read processing, mapping, and variant calling

Whole genome sequencing libraries were demultiplexed using the FASTQ Generation application available on the llumina BaseSpace Sequence Hub (basespace.illimina.com) and paired reads were quality trimmed using Trimmomatic v0.36 [22] with the following options: LEADING:20 TRAILING:20 MINLEN:75 AVGQUAL:20. Trimmed reads were mapped to the *S*. *japonicum* reference genome (ASM636876v1) [23] using default parameters in BWA [24] and reads were sorted using SAMtools for downstream analysis [25]. We called variants using two different methods to evaluate the potential impacts that variant discovery methods may have on inferences of fine-scale relatedness; the first method (BCFtools) is computationally faster and thus makes analysis of many samples computationally feasible, while the second method (GATK) is considered more robust yet is far more computationally intensive. First, we conducted variant calling using a combination of SAMtools ‘mpileup’ and BCFtools [26] by filtering our data to only include sites with a minimum coverage of 5x per sample using the flag -e ‘FORMAT/DP<5’ and sites which included quality scores above 30 (QUAL > 30). Second we called variants with GATK v.4.0.8.1 using the best practices workflow [27] by generating individual VCFs using ‘HaplotypeCaller’, specifying–ERC GVCF, and then calling population variants using ‘GenotypeGVCFs’. We then used GATK ‘VariantFiltration’ to further hard filter based on GATK’s best practices recommendation (QD < 2, QUAL < 30.0, SOR > 3.0, FS > 60.0, MQ < 40.0, MQRankSum < -12.5, ReadPosRankSum < -8.0), and used GATK ‘SelectVariants’ to include only variants that met all filtering thresholds. Sets of variants from both variant call approaches were further filtered to retain only biallelic SNPs from exonic regions and to only include variants that are present in at least 80% of individuals and with a minor allele frequency (MAF) of > 0.05 [11,28].

### Analysis of coverage across individuals

To assess the distribution of mapped read coverage across individuals, we examined coverage within exonic regions using SAMtools depth on all sites, including sites with zero coverage, and extracted exonic regions using BEDtools intersect [29] for the 67,109 annotated exons of the *S*. *japonicum* reference genome [23]. We specifically focused on analyses of coverage within exon regions because the *S*. *japonicum* genome contains a high fraction of repetitive DNA (~45%; [23]) that may lead to inaccurate inferences of read mapping and coverage. We expect exonic sequences to be mostly single- or low-copy sequences that should suffer the least amount of mapping error and inconsistency. In addition to calculating coverage from exonic regions, we also estimated coverage in 50kb windows across the entire genome using a combination of SAMtools depth and BEDtools map.

### Population genomic analyses, rare allele sharing, and posterior estimates of relatedness

To demonstrate the potential to use genome-scale data to infer relatedness among sampled miracidia sampled, we performed two sets of analyses. We used our BCFtools variant call set for all population genomic analyses to provide a direct comparison to analyses of down-sampled coverage experiments (see methods below), and because BCFtools made the variant calling of a large number of replicated datasets computationally feasible. First, we used clustering methods to evaluate the extent to which the genetic differences between samples corresponded with the spatial distribution of samples collected. Such methods can be used to examine the spatial scales over which infections are being acquired–allowing us to distinguish highly localized transmission patterns from evidence that infections are being acquired over larger spatial scales. We performed a principal component analysis (PCA) using the R package ‘adegenet’ [30] to visualize the spatial distribution of genetic variance between all individuals for exonic SNPs. Using the same dataset we also inferred relatedness among all samples by constructing a neighbor-joining tree from average pairwise genetic distances using the R package ‘ape’ [31]. We also calculated nucleotide diversity across all exonic regions using 1Kb windows using VCFtools [32]. To facilitate direct comparison between variant-calling methods, a neighbor-joining tree analysis was conducted on the GATK-called variant set.

Second, we estimated pairwise rare allele (MAF <0.1) sharing proportions (which can range from 0–1) to infer degrees of relatedness between all pairs of miracidia as in Shortt et al. [12] (code available at https://github.com/PollockLaboratory/Schisto) for both variant datasets. Estimates of near-relatedness have a range of applications such as allowing the ability to infer the number of adult mating pairs in a single host, the size of the schistosome population in a region, and the identity of potential infection sources [12]. To avoid overestimating relationships because of linked variants, we calculated the mean rare allele sharing across 2,000 replicates for a random sample of 3,000 variants from the exonic dataset. We estimated the posterior probability of each subsampled mean degree of relatedness between every pair of miracidia as in Shortt et al. [12]. For these calculations, we used allele sharing distributions for each degree of relatedness estimated by Shortt et al. [12] for *S*. *japonicum* sampled from the same geographic region. There are some differences between the samples used within Short et al. [12] and ones used within this study that may slightly affect the allele sharing distributions, most notably that Shortt et al. [12] used randomly sampled genomic loci whereas here we use exons. However, with 200 samples overall and from many more villages, Shortt et al. [12] had large numbers of unrelated individuals from large numbers of siblings sampled within hosts. Accordingly, we used the allele sharing distributions from Shortt et al. [12] to calculate posterior relatedness values for our data because we expect the mean and variance estimates from Shortt et al. [12] to be far more accurate than what we could calculate from our much smaller sample size.

### Downsampling to estimate impacts of lower coverage sequencing

Individual genomes in this study were sampled at relatively high coverage (Table 2; 18 out of 22 had over 100x exon coverage), but we were interested in whether future sampling of individuals could be accomplished at lower coverage; this would enable greater numbers of samples to be sequenced by reducing the cost per individual sample. To test this, we down-sampled our data to represent the equivalent of 35x, 30x, 20x, 15x, 10x, and 5x coverage per individual. We conducted 5 replicates of each coverage dataset to test the impacts that lower levels of sampling might have on estimates of genetic variation, on our analyses of clustering of individuals based on genetic differentiation, and on estimation of relatedness based on rare allele sharing. We calculated the proportion of reads needed to simulate each coverage dataset for each individual and used the ‘view–s’ flag in SAMtools to create new down-sampled bam files. We then used the methods described above to call and filter variants for all exonic regions. We only used our variant call set from BCFtools for down-sampled coverage analysis, as using GATK to generate a similar variant call set for all replicated coverages and samples would have required extreme computational time and was thus not feasible. We performed PCAs and estimated the proportion of shared rare alleles to infer how lower coverage affects the resolution of population genomic variation and inferences of relatedness. To test that estimates of rare allele sharing did not differ among replicated sampling of mapped reads for each level of coverage we performed a Kruskal-Wallis [33] test among distributions of estimated rare allele sharing (RAS) values for a given coverage level estimated from resampling of mapped reads. We then examined differences between the distributions of rare allele sharing derived from high-coverage (35x) and successively lower levels of coverage using Kruskal-Wallis tests between a single replicate from each coverage level compared to high-coverage (35x), followed by a post hoc Dunn’s test [34] to account for multiple comparisons. We also estimated the impacts of down-sampling on inferences of population genomic structure by calculating the difference of within-village variances (based on PCA analyses) derived from datasets with varying levels of coverage.

## Results

### Genomic sequencing, mapping, and coverage

We sequenced a total of 22 whole genome amplified miracidia samples collected in 2016 from two villages in Sichuan, China (Table 2). The raw sequences are available at NCBI’s short read archive under the BioProject ID PRJNA650045 (accession numbers SAMN15692267-SAMN15692288). The miracidia sampled included 16 from 10 hosts living in Village C and 6 from a single host in Village M. We recovered an average of 263 million raw reads per individual miracidia with Q-scores > 20 (a Q-score of 20 indicates a 1 in 100 probability of an incorrect base call). The average number of mapped reads across all individuals was 228 million, with 20 out of 22 individuals having > 80% mapped reads (average: 93%), and two individual samples having low frequencies of mapped reads (<22%; Table 2). Low frequencies of mapped reads corresponded to low coverage of exons (Table 2), and so the two individuals with low coverage were subsequently excluded from all further downstream analyses because they appeared to be low-quality. To assess read coverage distributions across the 20 remaining samples, we surveyed coverage within exonic regions (S2 and S3 Figs) and found the mean across these individuals for average coverage among exons was 251x; the median across these individuals for median exon coverage was 44x, and 22x for whole genome coverage (see Table 2 for additional information and summary statistics related to coverage across samples).

### Population genomic analyses, rare allele sharing and posterior estimates of relatedness

**Fig 1:**
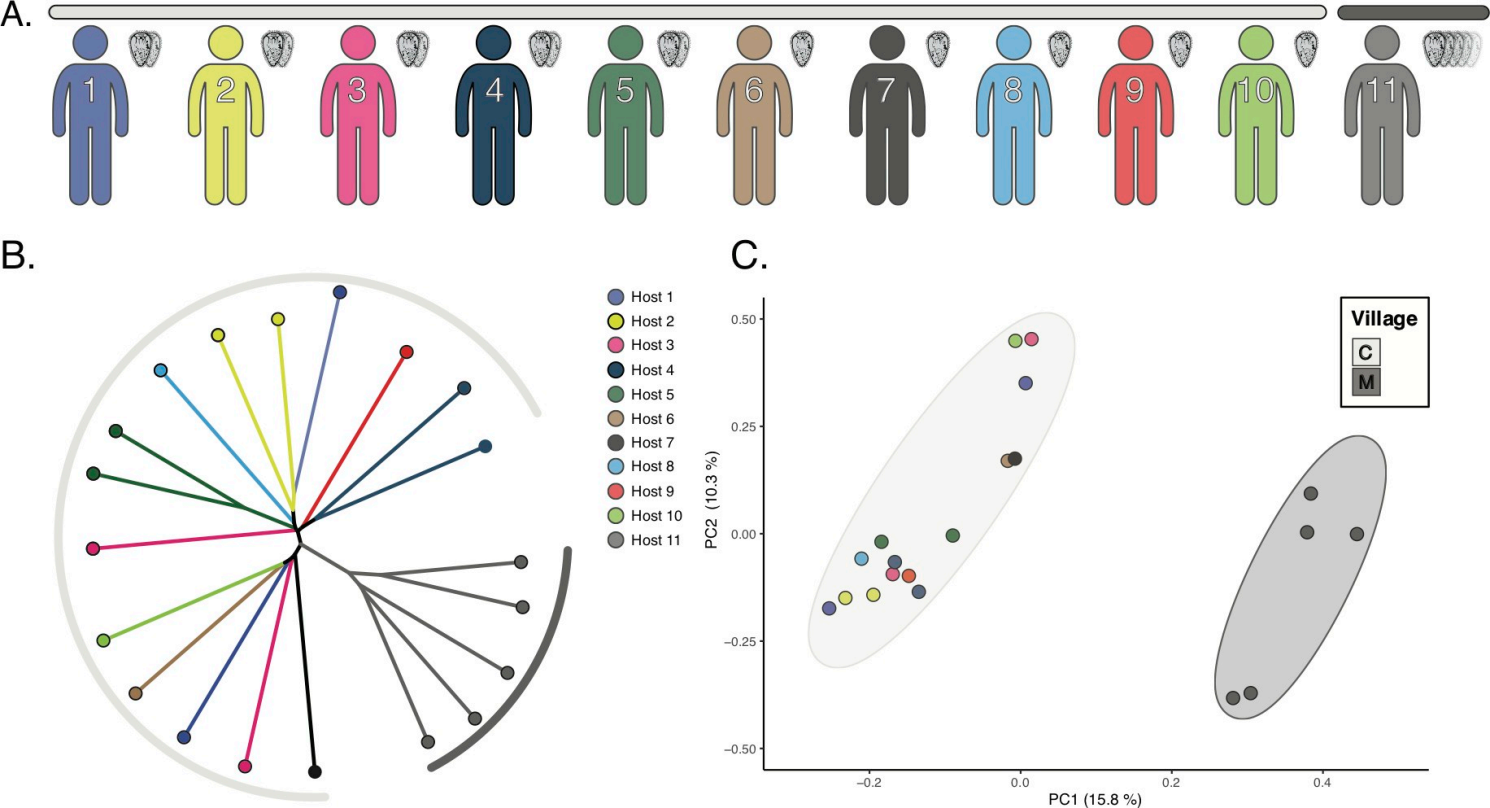
Overview of sampling and genetic separation between villages. A) Number of samples from each host with host identification labeled within colored human silhouettes. Village identification is indicated by the colored bars overhead that correspond to the figure legend in panel C. B) Neighbor Joining tree using all exonic variants from all miracidia, excluding the two samples with particularly low mapped read percentages. C) Principal Component Analysis of all exonic variants with the first principal component representing 15.8% of the variation and the second representing 10.3%.

Because of the high proportion of repetitive DNA found in the *S*. *japonicum* genome (~45%), we analyzed coverage within exons, which are low-copy and more likely to map consistently and confidently. Our BCFtools exonic variant dataset for the 20 remaining samples contained 429,827 SNPs, which was the equivalent of 2.4% of all exonic bases (17.8 Mb; S4 Fig), and our variant set using the GATK pipeline resulted in 369,827 SNPs (2.1% of exonic bases). Both variant calling pipelines resulted in a total of 258,665 common exonic SNPs that were shared between variant sets, along with 109,725 SNPs uniquely called by GATK, and and 171,162 SNPs uniquely called by BCFtools (S5 Fig). Our estimate of relatedness among samples based on neighbor-joining clustering for BCFtools variants indicated that samples from the two villages are not closely related and represent distinct genetic clusters (Fig 1), and this tree was similar to that based on GATK-called SNPs (S5D Fig). This analysis also indicated that the relative genetic similarity of miracidia within hosts is variable. Within village C (where there are samples from multiple hosts), we find that miracidia from the same host vary in relatedness, with some forming distinct host-specific clusters (e.g., host 5) and others representing relatively divergent lineages within the larger village C cluster (e.g., host 1; Fig 1B). The principal component analysis (PCA) of BCFtools variant data indicates similar patterns, and expose a clear genetic differentiation between samples from the two villages–this between-village distinction underlies much of the variation in PC1, which represented 15.8% of the variation among samples. The second principal component (PC2; explaining 10.3% of the variation) separated samples within villages, and like the neighbor-joining tree, highlights distinctions among miracidia within some hosts (e.g., host 3) and the similarity among miracidia in other hosts (Fig 1C).

**Fig 2:**
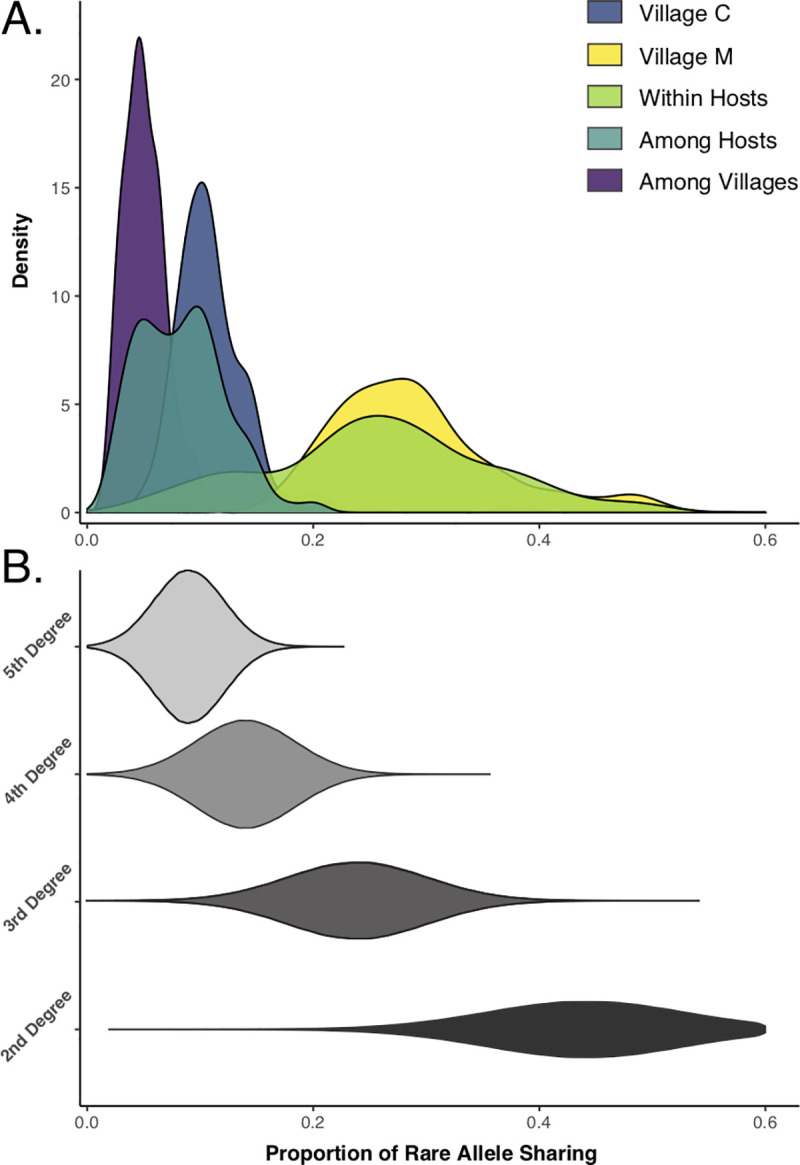
Rare allele sharing between all pairs of miracidia sampled. A) Proportions of rare alleles shared within and among villages and hosts. B) Posterior probabilities for different degrees of relatedness are indicated by height for different proportions of rare allele sharing for 2^nd^- 5^th^ degree relationships (panel adapted from (12)).

To infer degrees of relatedness among miracidial samples and assess the impacts of different variant calling pipelines on relatedness inferences, we first estimated patterns of rare allele sharing (RAS) among all pairs of sampled miracidia using both exonic SNP datasets to illustrate distributions of overall RAS values (S5C Fig), and values within and among hosts and villages (Fig 2A). Distributions of RAS values generated by BCFtools and GATK are different, with GATK-based RAS values being consistently lower than those based on BCFtools (S5C Fig and S1 and S2 Tables). To provide context to the distributions of allele sharing, Fig 2B shows posterior probability distributions for different degrees of relatedness from our BCFtools dataset (Y-axis) for various proportions of rare allele sharing (X-axis). Distributions of allele sharing across all samples indicate that relationships between miracidia vary widely within hosts, ranging from 2^nd^ degree (siblings) to 5^th^ degree relatives (second cousins). Within village C, where we sampled miracidia from multiple hosts, most relationships among sampled miracidia are 4^th^ degree (first cousin-level) and 5th degree. The single host that we sampled from village M shows patterns of between-miracidia allele sharing that we estimate to be 2^nd^ and 3^rd^ (avuncular/pibling, half-siblings, and double-first cousins) degree relationships. The distribution of allele sharing among all miracidia between villages indicates that more distant relationships link samples from the two adjacent villages, with most being 5^th^ degree or greater (less related or unrelated) (Fig 2).

**Fig 3:**
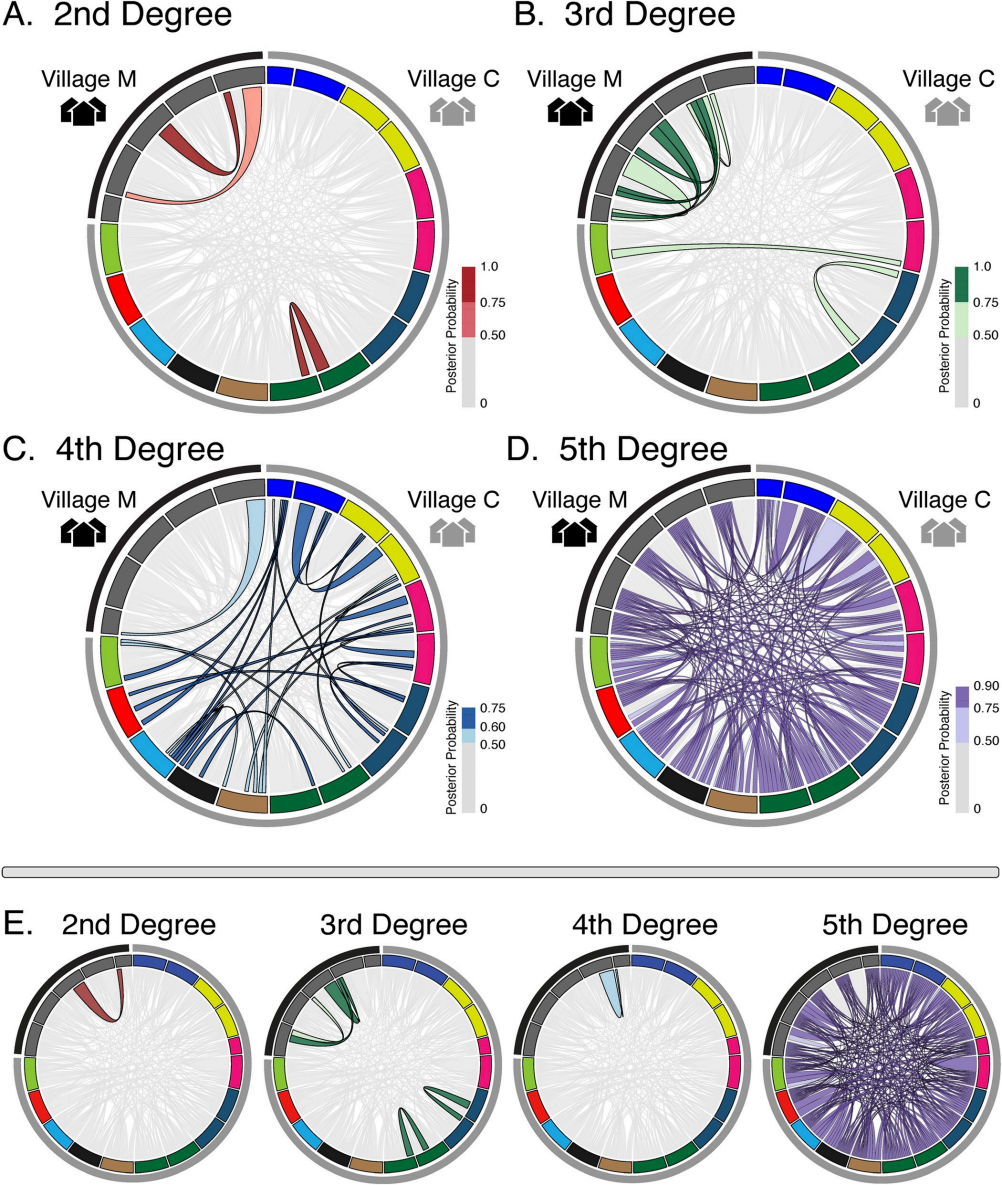
Posterior probability estimates of relatedness among sampled miracidia. Bands represent villages and miracidia are colored by host. All posterior probability estimates are shown in light gray, while colored ribbons represent relationships that have a posterior probability ≥0.50. A) Red ribbons representing 2^nd^ degree (full sibling) relationships among miracidia that have a posterior probability estimate ≥0.50 or higher. B) Green ribbons representing 3^rd^ degree (half-sibling or pibling or double-first cousin) relationships among miracidia that have a posterior probability estimate ≥0.50. C) Blue ribbons representing 4^th^ degree (first cousin) relationships among miracidia that have a posterior probability estimate ≥0.50. D) Purple ribbons representing 5^th^ degree relationship among miracidia. E) Posterior probability estimates of relatedness using the GATK exonic dataset.

To better understand detailed patterns of allele sharing in the context of relatedness, we estimated posterior probabilities for both exonic datasets of discrete degrees of pairwise relatedness among all sampled miracidia (Fig 3). To accommodate the inherent uncertainty in classifying miracidial pairs into discrete degrees of relatedness, we used a posterior probability approach that incorporates uncertainty when estimating relatedness and assigning miracidial pairs to particular degrees of relatedness. To highlight the relatedness structure among miracidial samples, we show relationships inferred at >50% posterior probability, and include various color shades to further indicate posterior probabilities of inferred relationships (Fig 3). Estimates of posterior probabilities of relatedness, based on both SNP datasets, suggest that there are multiple sibling and half-sibling pairs within each village, all of which share the same host (Fig 3A, 3B and 3E and S1 and S2 Tables). Further, no 2^nd^ degree relationships were inferred with high probability for miracidia pairs sampled from different hosts. In miracidia sampled from village C, we find that within hosts, miracidia are predominantly 4^th^ or 5^th^ degree relatives, suggesting that many of the hosts we sampled were infected by multiple (fairly distantly related) adult worm pairs. This pattern contrasts with the high degree of relatedness observed among miracidia from the host sampled from village M. We find that inferences of relatedness that are supported by lower posteriors tended to vary between estimates from the two datasets, whereas most higher-posterior inferences of relatedness tended to be conserved between inferences from these two datasets. There is also a general trend for results based on GATK variants to lead to inferences of slightly more distant relationships among miracidial pairs, compared to estimates from BCFtools variants; this trend is also consistent with lower RAS value distributions observed based on GATK variants (S5 Fig). However, both datasets inferred a number of conserved relationships with the highest degree of relatedness tending to be more conserved across datasets. We find that 82% of 5^th^ degree relationships were shared between methods and a total of 26% and 33% of 3^rd^ and 2^nd^ degree relationship respectively were conserved between miracidial pairs (Fig 3 and S3 Table). We did not observe any overlap in inferred 4^th^ degree relationships between datasets.

### Estimation of the relationship between coverage and population genomic inference resolution

**Fig 4:**
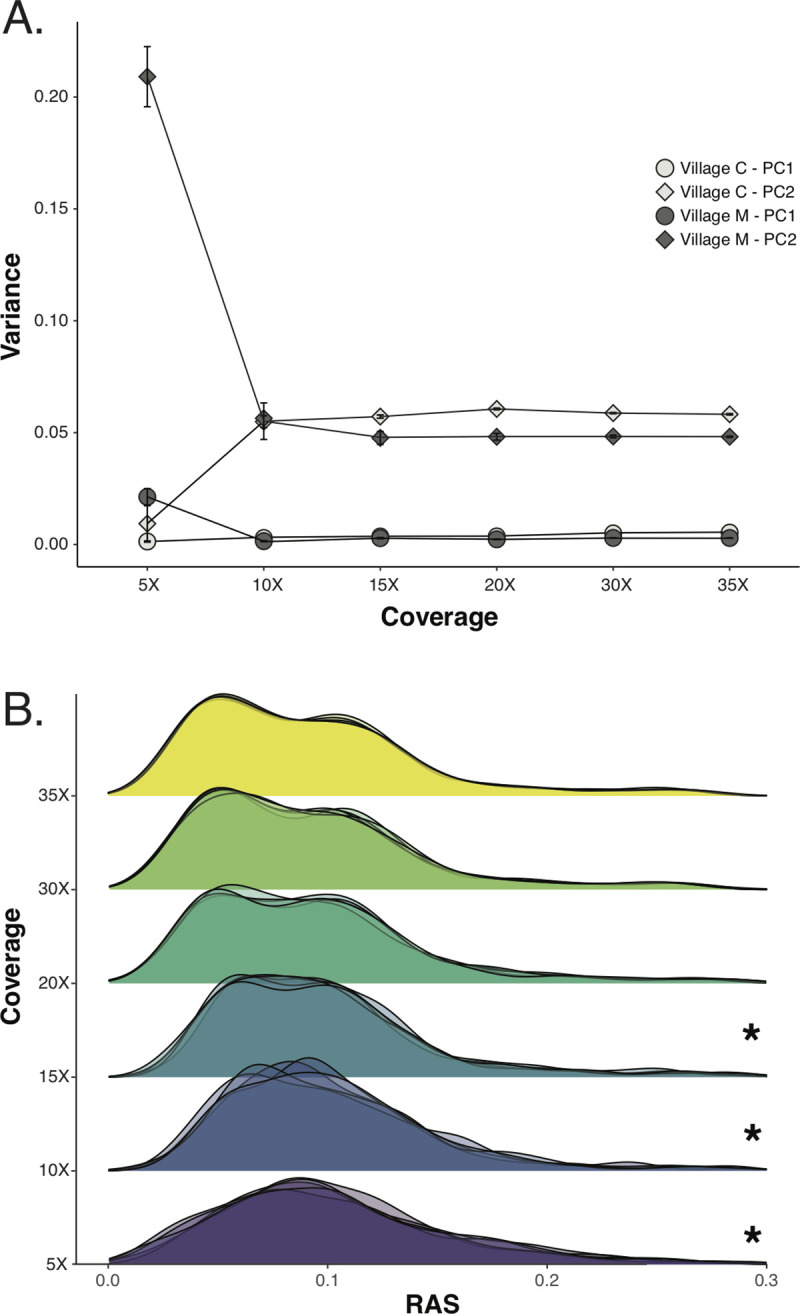
Effects of downsampling overall genomic coverage on estimates of the genetic variance and rare allele sharing across samples. A) Line graphs showing the differences in PC1 and PC2 variance for each village for all exonic variant downsampled datasets. For each coverage level, variances were estimated for each of five downsampled replicates; dots represent the mean variance across all replicates, with error bars indicating variance across replicates. B) Distributions of the proportion of rare alleles shared for all replicates for each downsampled coverage dataset. Coverage datasets that were significantly different from 35x are indicated by an asterisk. For each level of coverage, individual lines represent RAS distributions estimated from 5 replicates of resampling mapped reads.

To assess the degree to which lower coverage genomic datasets could detect comparable patterns of relatedness to those derived from our high-resolution WGS data, we down-sampled our dataset and compared resulting estimates of relatedness to our findings described above. Analyses of down-sampled datasets suggest that coverage levels as low as 15x produce remarkably similar inferences of spatial structuring and rare allele sharing to our full dataset (271x coverage), and thus would be expected to provide similarly accurate inferences of relatedness among samples (Figs 4, S6 and S7). To understand the impact that varying levels of coverage may have on discerning spatial patterns of genetic variation across samples, we conducted PCA analyses including all individuals using different coverage datasets. We then considered whether the relative variance in each village for the first two principal components, PC1 and PC2, were consistent for five replicates for each downsampled estimate. The within-village variances were highly consistent for 15x coverage and higher (Fig 4A), indicating that 15x coverage is sufficient to obtain good variance estimates. The amount of variance explained by PC loadings (for PC1 and PC2) increased with coverage (Figs 4A and S8). Similarly, the distributions of rare allele sharing also remained consistent at coverage levels down to approximately 10x and did not differ significantly above 15x coverage (p > 0.05 Kruskal-Wallis test), with coverage levels below this showing greater divergence in distributions of RAS (Figs 4B, S6 and S7). In Fig 4B, for a given coverage level, individual lines represent RAS distributions estimated from 5 replicates of resampled mapped reads, and in all cases, we found very little variation among replicates and this variation was not statistically significant based on a Kruskal-Wallis test.

### Exploration of exonic nucleotide diversity

To examine patterns of nucleotide diversity and identify genomic regions that show extremely high levels of diversity, we conducted sliding window (1kb) analyses of nucleotide diversity across exonic regions (S9 Fig). We identified the 10 genomic windows with the highest nucleotide diversity (>0.08), several of which were adjacent to other extreme windows, collectively forming six independent clustered regions (S9 Fig). Genes in these six regions are shown in Table 2 and include the gene *Paramyosin*, the target of a schistosomiasis vaccine under development [35].

## Discussion

### Population genomic inferences from individual archival miracidia

Despite the potential for high-resolution genomic approaches to resolve questions about schistosome parasite transmission and responses to control measures, high monetary costs and low per-individual DNA yields have limited the feasibility of collecting such population-level WGS datasets. Previous pooled-sample studies have, however, provided strong motivation to overcome these barriers and demonstrated the value of population-level whole genome sequencing of schistosomes to understand the genomic impacts of selection pressures on schistosome parasites [36,37]. While a valuable first step, such pooled-sample approaches lack critical resolution required to identify local transmission patterns, as well as within- and among-host infection patterns. Recently, we addressed one key barrier to progress in this area–the challenge of obtaining sufficient DNA for WGS from single schistosome individuals–by demonstrating the feasibility of using whole genome amplification (WGA) on archival miracidia, preserved on Whatman cards, to generate reduced representation genomic libraries of individual miracidia [11,12]. Here, we demonstrate that the generation of WGS data from individual archival miracidia is now both technically and economically feasible, thereby removing both major barriers that have prohibited the use of individual-scale population genomic information to understand detailed patterns of parasite relatedness and underlying parasite biology.

Our analyses illustrate the potential to sample individual archival miracidia with relatively low genome coverage while maintaining accurate population-level inferences of genetic variation, allowing large numbers of miracidia to be sampled at a reasonable cost. We tested the feasibility of whole genome sequencing of individual archival miracidia by sampling 22 miracidia from geographically close localities (~12 km). We purposefully sampled each individual at a high level of genomic coverage (mean exonic coverage >250x)–far higher than is typical for WGS genotyping studies (e.g., 10-40x)–to test the ability of WGS data to discern fine-scale patterns of relatedness and differentiation. This high-coverage genomic sampling enabled us to subsequently downsample our data to determine the degree of lower coverage sequencing that would be sufficient to produce comparable resolution and accuracy of inferences. We expect to have particularly high power to detect SNPs based on our high individual coverage, and this feature of our data likely explains our slightly higher SNP diversity estimates from both variant calling approaches compared to previous studies [36]. When considering how much data is required per individual, it is important to appreciate that different types of inferences based on WGS data may depend more or less on the accuracy of genotyping inferences, and thus on coverage obtained per individual. While there is substantial empirical and theoretical literature available that predicts the relationships between genotype uncertainty, coverage, and sequence quality [38,39], we conducted empirical estimates here because previous studies did not incorporate WGA, which has the potential to introduce additional error.

Our estimates suggest that lower levels of coverage, down to approximately 15x, produce estimates of population genetic structure and rare allele sharing that are statistically indistinguishable from those derived from analyses using much higher levels of genomic sequencing coverage. These inferences closely match estimates from the literature for expected decays in inferential power with sequencing depth (e.g., [38,39]), which suggests that the additional WGA step in our approach is not likely to introduce substantial error. These findings highlight the potential to sequence large numbers of individual miracidium genomes at low-to-moderate coverage without compromising the accuracy of downstream inferences of relatedness between schistosomes collected at fine spatial scales. Furthermore, with whole genome data, even lower per-sample coverage may be sufficient for inferences that are less sensitive to genotyping errors, including relatedness, population structure, and various genomic scans [40].

### Impacts of variant calling on inferences of fine-scale measures of relatedness and paths to future refinement

The potential impacts of different analytical approaches on inferences of relatedness is important to understand and consider when making downstream biological conclusions. One potential source of inference variation is the impact that different variant calling pipelines may have on subsequent population genomic inferences [41]. Here, we compared two variant calling approaches (BCFtools versus GATK) to reveal modest differences in the distributions of inferred rare allele sharing that translated to slight differences in inferred posterior estimates of relatedness. Overall, the GATK variant dataset inferred lower levels of allele sharing between individuals (S5A and S5B Fig) and thus resulted in greater frequencies of low-level relationships within villages and hosts (i.e., 2^nd^ and 3^rd^ degree) compared to similar estimates derived from the BCFtools variant dataset. One possible explanation for higher proportions of rare allele sharing based on BCFtools is the greater overall number of SNPs that resulted from BCFtools (S5 Fig). This greater number of BCFtools SNPs may capture more instances in which miracidia pairs share rare-alleles, and may therefore lead to inferences of lower degree (closer) relationships between individuals.

Future efforts to further investigate how various SNP calling pipelines and filtering schemes may impact inferences of relatedness would be valuable for developing strategies for increased accuracy and consistency. One advantage of our approach that uses posterior probabilities [42] is that relatedness inferences can readily incorporate uncertainty, and indeed differences in estimates of relatedness that tended to vary between SNP datasets tended to be those associated with lower posterior probabilities. As larger sample size genomic datasets become increasingly available, recalibration of posterior estimation of relatedness based on larger sample sizes will further improve the accuracy of this approaches. A source of variation not accounted for in our current approach is the impact of sex-linked regions and sex chromosomes on estimation of rare allele sharing and relatedness; for example female-female sibling pairs should share slightly more rare alleles than male-female siblings because the same-sex pair shares the same sex chromosome genotype. Accordingly, revised approaches that identify the sex of sampled individuals and account for sex-linked loci in the estimation of rare allele sharing would further improve the accuracy and precision of inferences of relatedness.

### Fine scale patterns of relatedness within and between villages

Our analyses of a limited sampling of miracidia and hosts indicate that individual miracidia WGS can provide insight into regional patterns of infection. Broadly, our inferences of relatedness and diversity between villages indicate that infection sources tend to be local (i.e. village specific), in agreement with recent research based on ddRADseq data from the same region [12]. We found that despite the close proximity of the two villages sampled (~12 km), miracidia are more closely related to other individual parasites within the same village than they are to parasites in a nearby village (although village M was only represented by 5 miracidia from a single human host). This finding again is consistent with that from ddRADseq data from the same region [12]. These results demonstrate that individual WGS sampling can powerfully identify local patterns of infection that are relevant for understanding transmission and guiding control efforts.

Comparing patterns of miracidia diversity and relatedness within hosts can also provide insight into the relative number of infection events that have contributed to observed infections in a population. For example, miracidia from individual hosts in village C exhibit a range of familial relationships (3^rd^-5^th^ degree/unrelated; Fig 3B–3E), suggesting that multiple unique infection events have led to these infections. In contrast, most miracidia sampled from a single human host in village M exhibit closer familial relationships (2^nd^ and 3^rd^ degree; Fig 3A–3C and 3E). While village M is only represented by a single host, the pattern of low degrees of relatedness within this single individual is in stark contrast to the higher degrees of miracidia relationships seen in multiple hosts from village C (Fig 3C–3E). Combining systematic sample collection with estimates of familial relationships between parasites is thus a valuable tool for identifying and comparing the number of transmission events driving infection in different locations.

Our results further demonstrate the utility of this approach by making inferences about the number of adult parental worms infecting a single host, and how this number varies between hosts and villages. The paucity of miracidia inferred to be siblings and half-siblings (2^nd^ and 3^rd^ degree relatives) in village C indicates multiple mating worm pairs in each sampled village C host (Fig 3A, 3B and 3E). The relationships inferred from all individuals within this village further indicate that local re-infection may be common, and this is supported by multiple hosts having miracidia most closely related to those from other hosts within the same village (Fig 3D and 3E). For example, we sampled two miracidia each from three hosts (i.e., hosts 1–3; Fig 3B and 3E) and found that all individual miracidia were more closely related to individuals from other hosts, but varied in the highest degree of relatedness (3^rd^, 4^th^, and 5^th^ degree relationships; Fig 3B–3E). We also show that the majority of connections between all miracidia within this village are 5^th^ degree relationships, suggesting these hosts may share a local network of transmission. The mechanisms underlying these contrasting dynamics might be driven by access of miracidia to their intermediate host (a freshwater snail, *Oncomelania*), or other factors [43,44]. While the scope of this study is not sufficient to differentiate these factors, our results demonstrate how such high-resolution inferences can broadly inform and prioritize control measures by identifying differences in infection parameters across hosts and host populations.

Our preliminary analyses of population genomic variation further highlight the potential value of such data for understanding schistosomiasis treatment strategies, particularly once larger-scale sampling becomes available. Of the regions we identified with extreme levels of nucleotide diversity, one contained the *Paramyosin* gene, which has previously been identified as a promising vaccine candidate for both *S*. *japonicum* and *S*. *mansoni* (Table 3; [34,42]). Previous experimental work has shown that mice vaccinated with Paramyosin exhibit increased resistance to cercaria (the free-swimming life stage which infects the definitive host; [43]), and the presence of natural Paramyosin antibodies in humans was found to predict resistance to *S*. *japonicum* re-infection [45–47]. Our finding of high genetic diversity at the Paramyosin locus of schistosomes suggest that this specific locus may be under diversifying selection, potentially in response to natural antibodies in regional hosts. While preliminary, this finding in *Paramyosin* provides two potential examples for how understanding schistosome genomic variation may help inform treatment. First, if *Paramyosin* is targeted as an antibody therapy, understanding natural population variation in this gene is important for developing broadly effective antibodies. Second, the finding of similarly high diversity in other loci may be indicative of other genes that exhibit important, and likely poorly-understood, interactions with host genomes. Thus, similar but expanded WGS studies hold promise for unlocking new insight into host-parasite interactions.

**Table 3:** Genes found within the top 10 windows (1kb) of nucleotide diversity. Scaffold number, gene product, accession numbers, and nucleotide diversity are reported.

Scaffold	Product	NCBI Accession	Nucleotide Diversity
SKCS01000014.1	Aldehyde dehydrogenase X isoform 1	TNN21037.1	0.088
SKCS01000088.1	Hypothetical Protein	TNN17749.1	0.162
SKCS01000138.1	GPI ethanolamine phosphate transferase 3 isofrom 2	TNN15975.1	0.111
SKCS01000192.1	Myosin catalytic light chain LC mantle muscle isoform 2	TNN13420.1	0.121
SKCS01000199.1	NAD(P) H-hydrate epimerase isoform 3	TNN13111.1	0.091
SKCS01000434.1	Paramyosin isoform 1	TNN07303.1	0.157

### Future directions for schistosome population genomics

Population genomic approaches have the potential to answer previously inaccessible questions regarding the transmission of schistosomes and other human helminths during disease control efforts and the impact of these efforts on parasite biology, information that can be used to improve current and future disease control efforts. China in particular has pursued one of the most aggressive and long standing schistosomiasis control programs in the world, starting in the 1950s and re-invigorated recently through a multi-pronged campaign to eliminate schistosomiasis nationwide [48,49]. In the Sichuan region, for example, control activities have included over a decade of both mass and targeted chemotherapy in humans and bovines, as well as snail control and other environmental modifications [50,51]. The analyses presented here and our prior work indicate that humans typically acquire infections from local sources and that infections are sometimes retained despite host treatment [12]. These findings underscore the value of archival miracidial WGS approaches for elucidating transmission pathways in areas where the disease has persisted despite control efforts.

Moreover, the methods presented here can be used to assess patterns of selection on parasite genomes imposed by decades-long control measures. Previous evidence for drug resistance in other schistosome systems [52] has been used as a basis for shifting treatment regimens [53]. The ability to sequence individual whole genomes on a population scale can provide a powerful means for detecting evidence of selection-driven drug resistance in parasite genomes that can inform treatment strategies and potentially provide early warning signs of drug-resistant parasite lineages. Future WGS studies with larger sample sizes have the potential to leverage more detailed analysis of genomic diversity across temporal and spatial dimensions that may be particularly informative for interpreting patterns of past and ongoing transmission, as well as shifts in parasite biology linked to selection-driven genomic changes in parasite populations. Our results support the practical and economic feasibility of such large-scale population genomic studies on schistosomes, and demonstrate the potential to use archived samples to provide key longitudinal sampling.

## Supporting information

S1 Table Pairwise comparison showing rare allele sharing values and posterior probabilities for each degree of relatedness for SNPs called using BCFtools. (XLSX)

S2 Table Pairwise comparison showing rare allele sharing values and posterior probabilities for each degree of relatedness for SNPs called using GATK. (XLS)

S3 Table Total numbers of each inferred degree of relatedness between variant calling and overlap between the two methods. (DOCX)

S1 Fig Map showing geographic localities and straight-line distance of sampled villages from Sichuan Province, China. The villages are separated by 12 km (Euclidean distance) or 17 km by roads (many of which are too small to be indicated on this map).
(TIF)

S2 Fig Coverage distribution across all exons for all samples used with the x-axis extending to a maximum coverage of 250x. Each color represents a single miracidium sample.
(TIF)

S3 Fig Plot showing the median coverage within 1Kb windows for all samples across exonic regions. (TIF)

S4 Fig Histogram of SNP quality scores for all retained exonic SNPs after filtering. The red bar denotes the minimum quality SNP score of 30.
(TIF)

S5 Fig Comparisons of variant calling methods. A) Venn diagram showing the number of exonic SNPs that were specific to each variant calling pipeline and the overlap between each method. B) Venn diagram showing the number of rare allele variants specific and shared between each variant calling pipeline. C) Rare allele sharing distributions for each variant calling pipeline. D) Neighbor-joining tree for all exonic variants called using GATK.
(TIF)

S6 Fig Effects of downsampling on overall genomic coverage on distribution of the overall proportion of shared rare alleles (RAS). Panels A-F show the distribution of RAS values between pairs of samples from exonic variants for among and between villages/hosts of all downsampled coverage datasets. Colors correspond to the same categories labeled in Fig 2 of the main text.
(TIF)

S7 Fig Effects of downsampling on overall patterns of rare allele sharing. Panels A-F show pairwise comparisons of rare allele sharing for all downsampled coverage datasets with village identity highlighted by the colored bars above.
(TIF)

S8 Fig Effects of downsampling on estimates of the genetic variance and rare allele sharing among samples. Panels A-F show principal component analysis (PCA) of simulated downsampled coverage. Overall percentage of explained variance are noted next to each PC axis
(TIF)

S9 Fig Nucleotide diversity within 1Kb windows (n = 46582) across exons from the full coverage variant dataset. The ten windows with the highest nucleotide diversity are indicated by red dots, and all other values of nucleotide diversity are indicated with gray dots.
(TIF)

## References

[1] WHO. Accelerating work to overcome the global impact of neglected tropical diseases–A roadmap for implementation. Geneva: WHO; 2012.

[2] Hampton T. Collaborative effort targets 17 tropical diseases for control, elimination. JAMA. 2012;307(8):772 10.1001/jama.2012.201 22357823

[3] Hotez PJ, Fenwick A, Savioli L, Molyneux DH. Rescuing the bottom billion through control of neglected tropical diseases. The Lancet. 2009;373(9674):1570–5. 10.1016/S0140-6736(09)60233-6 19410718

[4] Molyneux DH, Savioli L, Engels D. Neglected tropical diseases: progress towards addressing the chronic pandemic. The Lancet. 2017;389(10066):312–25. 10.1016/S0140-6736(16)30171-4 27639954

[5] Rudge JW, Lu D, Fang G, Wang T, Basanez M, Webster JP. Parasite genetic differentiation by habitat type and host species: molecular epidemiology of *Schistosoma japonicum* in hilly and marshland areas of Anhui Province, China. Mol Ecol. 2009;18(10):2134–47. 10.1111/j.1365-294X.2009.04181.x 19389178

[6] Barbosa LM, Silva LK, Reis EA, Azevedo TM, Costa JM, Blank WA, et al Characteristics of the human host have little influence on which local *Schistosoma mansoni* populations are acquired. PLoS Negl Trop Dis. 2013;7(12):e2572 10.1371/journal.pntd.0002572 24340115 PMC3854954

[7] Prugnolle F, Théron A, Pointier JP, Jabbour-Zahab R, Jarne P, Durand P, et al Dispersal in a parasitic worm and its two hosts: consequence for local adaptation. Evolution. 2005;59(2):296–303. 15807416

[8] Gower CM, Gouvras AN, Lamberton PHL, Deol A, Shrivastava J, Mutombo PN, et al Population genetic structure of *Schistosoma mansoni* and *Schistosoma haematobium* from across six sub-Saharan African countries: Implications for epidemiology, evolution and control. Acta Trop. 2013;128(2):261–74. 10.1016/j.actatropica.2012.09.014 23041540

[9] Steinauer ML, Agola LE, Mwangi IN, Mkoji GM, Loker ES. Molecular epidemiology of *Schistosoma mansoni*: a robust, high-throughput method to assess multiple microsatellite markers from individual miracidia. Infect Genet Evol. 2008;8(1):68–73. 10.1016/j.meegid.2007.10.004 18329981 PMC2278012

[10] Peterson BK, Weber JN, Kay EH, Fisher HS, Hoekstra HE. Double digest RADseq: an inexpensive method for de novo SNP discovery and genotyping in model and non-model species. PloS One. 2012;7(5):e37135 10.1371/journal.pone.0037135 22675423 PMC3365034

[11] Shortt JA, Card DC, Schield DR, Liu Y, Zhong B, Castoe TA, et al Whole genome amplification and reduced-representation genome sequencing of *Schistosoma japonicum* miracidia. PLoS Negl Trop Dis. 2017 1 20;11(1). 10.1371/journal.pntd.0005292 28107347 PMC5287463

[12] Shortt JA, Timm LE, Hales NR, Nikolakis ZL, Schield DR, Perry BW, et al Population genomic analyses of schistosome parasites highlight critical challenges facing end-game elimination efforts. bioRxiv. 2020; 10.1038/s41598-021-86287-y PMC7994584 33767307

[13] Le Clec’h W, Chevalier FD, McDew-White M, Allan F, Webster BL, Gouvras AN, et al Whole genome amplification and exome sequencing of archived schistosome miracidia. Parasitology. 2018;145(13):1739–47. 10.1017/S0031182018000811 29806576 PMC6193844

[14] Yin M, Liu X, Xu B, Huang J, Zheng Q, Yang Z, et al Genetic variation between *Schistosoma japonicum* lineages from lake and mountainous regions in China revealed by resequencing whole genomes. Acta Trop. 2016;161:79–85. 10.1016/j.actatropica.2016.05.008 27207135

[15] Wit J, Gilleard JS. Resequencing helminth genomes for population and genetic studies. Trends Parasitol. 2017;33(5):388–99. 10.1016/j.pt.2017.01.009 28319011

[16] Platt RN, McDew-White M, Le Clec’h W, Chevalier FD, Allan F, Emery AM, et al Ancient hybridization and adaptive introgression of an invadolysin gene in schistosome parasites. Mol Biol Evol. 2019;36(10):2127–42. 10.1093/molbev/msz154 31251352 PMC6759076

[17] Crellen T, Allan F, David S, Durrant C, Huckvale T, Holroyd N, et al Whole genome resequencing of the human parasite *Schistosoma mansoni* reveals population history and effects of selection. Sci Rep. 2016;6:20954 10.1038/srep20954 26879532 PMC4754680

[18] Carlton EJ, Bates MN, Zhong B, Seto EYW, Spear RC. Evaluation of mammalian and intermediate host surveillance methods for detecting schistosomiasis reemergence in southwest China. PLoS Negl Trop Dis. 2011;5(3):e987 10.1371/journal.pntd.0000987 21408127 PMC3050915

[19] Carlton EJ, Hubbard A, Wang S, Spear RC. Repeated Schistosoma japonicum infection following treatment in two cohorts: evidence for host susceptibility to helminthiasis? PLoS Negl Trop Dis. 2013;7(3):e2098 10.1371/journal.pntd.0002098 23505589 PMC3591324

[20] Liang S, Yang C, Zhong B, Qiu D. Re-emerging schistosomiasis in hilly and mountainous areas of Sichuan, China. Bull World Health Organ. 2006;84:139–44. 10.2471/blt.05.025031 16501732 PMC2626530

[21] Xiao N, Remais J V, Brindley PJ, Qiu D-C, Carlton EJ, Li R-Z, et al Approaches to genotyping individual miracidia of *Schistosoma japonicum*. Parasitol Res. 2013;112(12):3991–9. 10.1007/s00436-013-3587-9 24013341 PMC3834234

[22] Bolger AM, Lohse M, Usadel B. Trimmomatic: a flexible trimmer for Illumina sequence data. Bioinformatics. 2014;30(15):2114–20. 10.1093/bioinformatics/btu170 24695404 PMC4103590

[23] Luo F, Yin M, Mo X, Sun C, Wu Q, Zhu B, et al An improved genome assembly of the fluke *Schistosoma japonicum*. PLoS Negl Trop Dis. 2019;13(8). 10.1371/journal.pntd.0007612 31390359 PMC6685614

[24] Li H, Durbin R. Fast and accurate short read alignment with Burrows–Wheeler transform. Bioinformatics. 2009;25(14):1754–60. 10.1093/bioinformatics/btp324 19451168 PMC2705234

[25] Li H, Handsaker B, Wysoker A, Fennell T, Ruan J, Homer N, et al The sequence alignment/map format and SAMtools. Bioinformatics. 2009;25(16):2078–9. 10.1093/bioinformatics/btp352 19505943 PMC2723002

[26] Li H. A statistical framework for SNP calling, mutation discovery, association mapping and population genetical parameter estimation from sequencing data. Bioinformatics. 2011;27(21):2987–93. 10.1093/bioinformatics/btr509 21903627 PMC3198575

[27] Van der Auwera GA, Carneiro MO, Hartl C, Poplin R, Del Angel G, Levy-Moonshine A, et al From FastQ data to high-confidence variant calls: the genome analysis toolkit best practices pipeline. Curr Protoc Bioinforma. 2013;43(1):11–10. 10.1002/0471250953.bi1110s43 25431634 PMC4243306

[28] Schield DR, Adams RH, Card DC, Perry BW, Pasquesi GM, Jezkova T, et al Insight into the roles of selection in speciation from genomic patterns of divergence and introgression in secondary contact in venomous rattlesnakes. Ecol Evol. 2017;7(11):3951–66. 10.1002/ece3.2996 28616190 PMC5468163

[29] Quinlan AR, Hall IM. BEDTools: a flexible suite of utilities for comparing genomic features. Bioinformatics. 2010;26(6):841–2. 10.1093/bioinformatics/btq033 20110278 PMC2832824

[30] Jombart T. adegenet: a R package for the multivariate analysis of genetic markers. Bioinformatics. 2008;24(11):1403–5. 10.1093/bioinformatics/btn129 18397895

[31] Paradis E, Schliep K. ape 5.0: an environment for modern phylogenetics and evolutionary analyses in R. Bioinformatics. 2018;35(3):526–8. 10.1093/bioinformatics/bty633 30016406

[32] Danecek P, Auton A, Abecasis G, Albers CA, Banks E, DePristo MA, et al The variant call format and VCFtools. Bioinformatics. 2011;27(15):2156–8. 10.1093/bioinformatics/btr330 21653522 PMC3137218

[33] Kruskal WH, Wallis WA. Use of ranks in one-criterion variance analysis. J Am Stat Assoc. 1952;47(260):583–621.

[34] Dunn OJ. Multiple comparisons using rank sums. Technometrics. 1964;6(3):241–52.

[35] Tebeje BM, Harvie M, You H, Loukas A, McManus DP. Schistosomiasis vaccines: where do we stand? Parasit Vectors. 2016;9(1):528 10.1186/s13071-016-1799-4 27716365 PMC5045607

[36] Young ND, Chan K-G, Korhonen PK, Chong TM, Ee R, Mohandas N, et al Exploring molecular variation in *Schistosoma japonicum* in China. Sci Rep. 2015;5:17345 10.1038/srep17345 26621075 PMC4664899

[37] Li Y, Yin M, Wu Q, McManus DP, Blair D, Li H, et al Genetic diversity and selection of three nuclear genes in *Schistosoma japonicum* populations. Parasit Vectors. 2017;10(1):87 10.1186/s13071-017-2033-8 28212676 PMC5316221

[38] Nielsen R, Paul JS, Albrechtsen A, Song YS. Genotype and SNP calling from next-generation sequencing data. Nat Rev Genet. 2011;12(6):443 10.1038/nrg2986 21587300 PMC3593722

[39] Davey JW, Hohenlohe PA, Etter PD, Boone JQ, Catchen JM, Blaxter ML. Genome-wide genetic marker discovery and genotyping using next-generation sequencing. Nat Rev Genet. 2011;12(7):499–510. 10.1038/nrg3012 21681211

[40] Martin SH, Dasmahapatra KK, Nadeau NJ, Salazar C, Walters JR, Simpson F, et al Genome-wide evidence for speciation with gene flow in *Heliconius* butterflies. Genome Res. 2013;23(11):1817–28. 10.1101/gr.159426.113 24045163 PMC3814882

[41] Hwang S, Kim E, Lee I, Marcotte EM. Systematic comparison of variant calling pipelines using gold standard personal exome variants. Sci Rep. 2015;5(1):1–8. 10.1038/srep17875 26639839 PMC4671096

[42] Dunson DB. Commentary: practical advantages of Bayesian analysis of epidemiologic data. Am J Epidemiol. 2001;153(12):1222–6. 10.1093/aje/153.12.1222 11415958

[43] Li S-Z, Wang Y-X, Yang K, Liu Q, Wang Q, Zhang Y, et al Landscape genetics: the correlation of spatial and genetic distances of *Oncomelania hupensis*, the intermediate host snail of *Schistosoma japonicum* in mainland China. Geospatial Health. 2009;221–31. 10.4081/gh.2009.222 19440964

[44] Maszle D, Whitehead P, Johnson R, Spear R. Hydrological studies of schistosomiasis transport in Sichuan Province, China. Sci Total Environ. 1998;216(3):193–203. 10.1016/s0048-9697(98)00152-1 9646528

[45] Jiz MA, Wu H, Olveda R, Jarilla B, Kurtis JD. Development of paramyosin as a vaccine candidate for schistosomiasis. Front Immunol. 2015;6:347 10.3389/fimmu.2015.00347 26257728 PMC4508564

[46] Ramirez BL, Kurtis JD, Wiest PM, Arias P, Aligui F, Acosta L, et al Paramyosin: a candidate vaccine antigen against *Schistosoma japonicum*. Parasite Immunol. 1996;18(1):49–52. 10.1046/j.1365-3024.1996.d01-4.x 9223156

[47] Leenstra T, Acosta LP, Wu H-W, Langdon GC, Solomon JS, Manalo DL, et al T-helper-2 cytokine responses to Sj97 predict resistance to reinfection with *Schistosoma japonicum*. Infect Immun. 2006;74(1):370–81. 10.1128/IAI.74.1.370-381.2006 16368992 PMC1346663

[48] Wang L-D, Chen H-G, Guo J-G, Zeng X-J, Hong X-L, Xiong J-J, et al A strategy to control transmission of *Schistosoma japonicum* in China. N Engl J Med. 2009;360(2):121–8. 10.1056/NEJMoa0800135 19129526

[49] Xu J, Steinman P, Maybe D, Zhou X-N, Lv S, Li S-Z, et al Evolution of the national schistosomiasis control programmes in the People’s Republic of China. In: Advances in parasitology. Elsevier; 2016 p. 1–38. 10.1016/bs.apar.2016.02.001 27137441

[50] Liu Y, Zhou Y-B, Li R-Z, Wan J-J, Yang Y, Qiu D-C, et al Epidemiological features and effectiveness of schistosomiasis control programme in mountainous and hilly region of the People’s Republic of China. In: Advances in parasitology. Elsevier; 2016 p. 73–95. 10.1016/bs.apar.2016.02.019 27137443

[51] Li Y-S, Raso G, Zhao Z-Y, He Y-K, Ellis MK, McManus DP. Large water management projects and schistosomiasis control, Dongting Lake region, China. Emerg Infect Dis. 2007;13(7):973 10.3201/eid1307.060848 18214167 PMC2878251

[52] Cioli D, Botros SS, Wheatcroft-Francklow K, Mbaye A, Southgate V, Tchuenté L-AT, et al Determination of ED50 values for praziquantel in praziquantel-resistant and-susceptible Schistosoma mansoni isolates. Int J Parasitol. 2004;34(8):979–87. 10.1016/j.ijpara.2004.05.001 15217737

[53] Valentim CL, Cioli D, Chevalier FD, Cao X, Taylor AB, Holloway SP, et al Genetic and molecular basis of drug resistance and species-specific drug action in schistosome parasites. Science. 2013;342(6164):1385–9. 10.1126/science.1243106 24263136 PMC4136436

