# Supplementary material for: Patterns of relatedness and genetic diversity inferred from whole genome sequencing of archival blood fluke miracidia (Schistosoma japonicum)
Source: PLoS Negl Trop Dis. 2021 Jan 6;15(1):e0009020. doi: 10.1371/journal.pntd.0009020 (PMC7815185; doi:10.1371/journal.pntd.0009020)
Supplement: S3 Table — (DOCX) [file pntd.0009020.s003.docx]

**Table S3. Total numbers of each inferred degree of relatedness between variant calling and overlap between the two methods.**

| Familial Relationship (Highest Posterior Probability) | GATK | BCFtools | Shared |
| --- | --- | --- | --- |
| 2nd Degree | 1 | 3 | 1 |
| 3rd Degree | 5 | 10 | 4 |
| 4th Degree | 1 | 26 | 0 |
| 5th Degree | 183 | 151 | 151 |
