# Supplementary figures and images for: Patterns of relatedness and genetic diversity inferred from whole genome sequencing of archival blood fluke miracidia (Schistosoma japonicum)
Source: PLoS Negl Trop Dis. 2021 Jan 6;15(1):e0009020. doi: 10.1371/journal.pntd.0009020 (PMC7815185; doi:10.1371/journal.pntd.0009020)

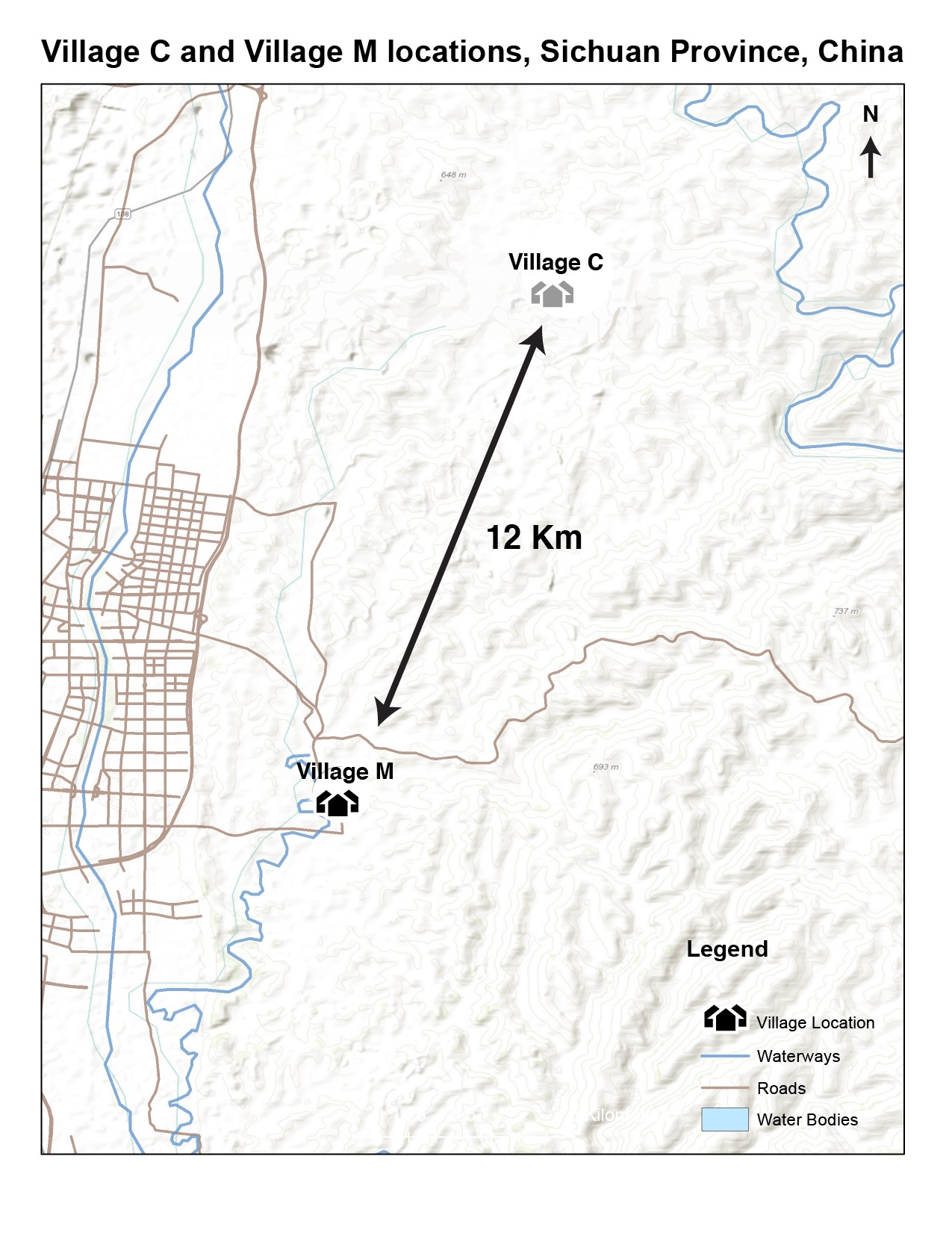

Supplement: S1 Fig — The villages are separated by 12 km (Euclidean distance) or 17 km by roads (many of which are too small to be indicated on this map). (TIF) [file pntd.0009020.s004.tif]

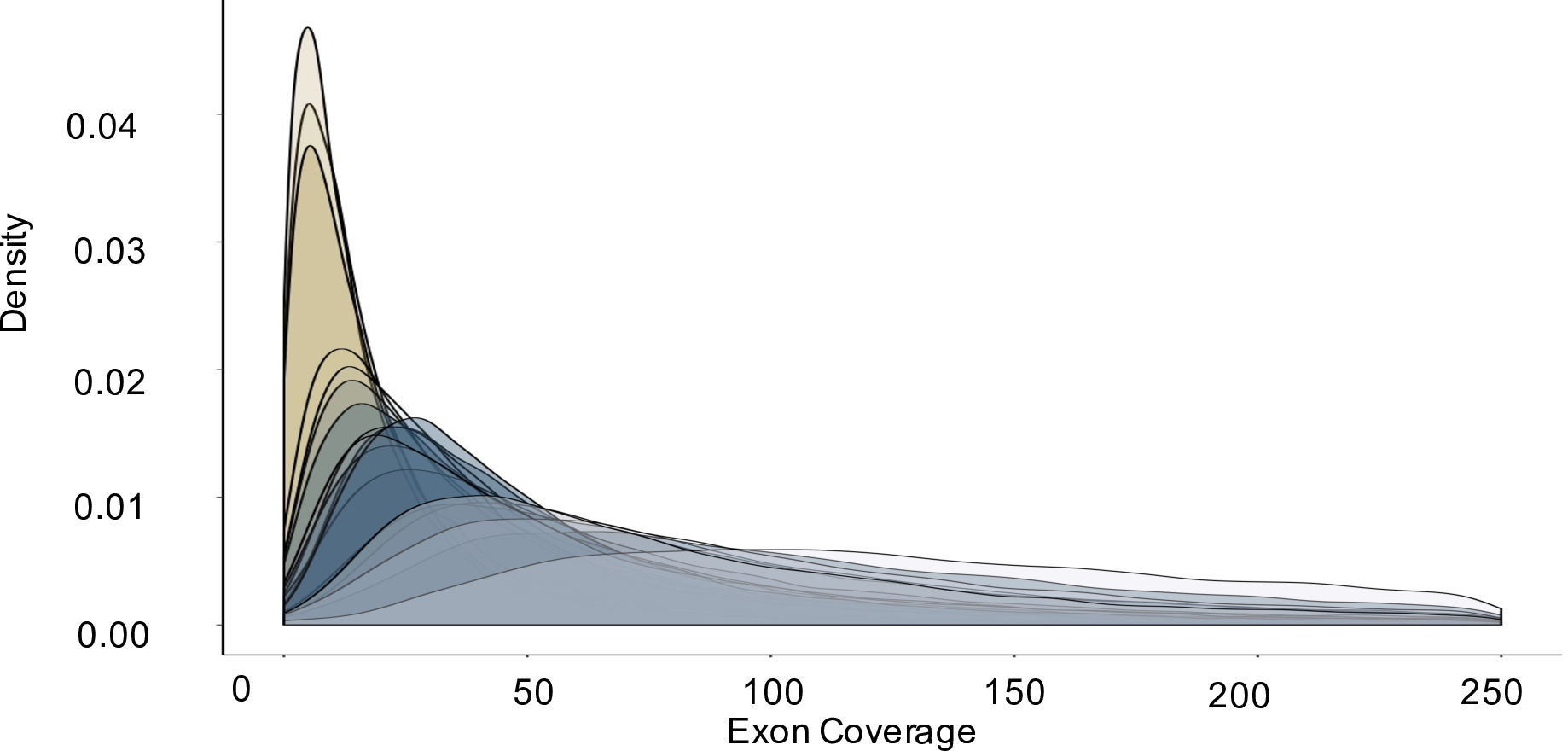

Supplement: S2 Fig — Each color represents a single miracidium sample. (TIF) [file pntd.0009020.s005.tif]

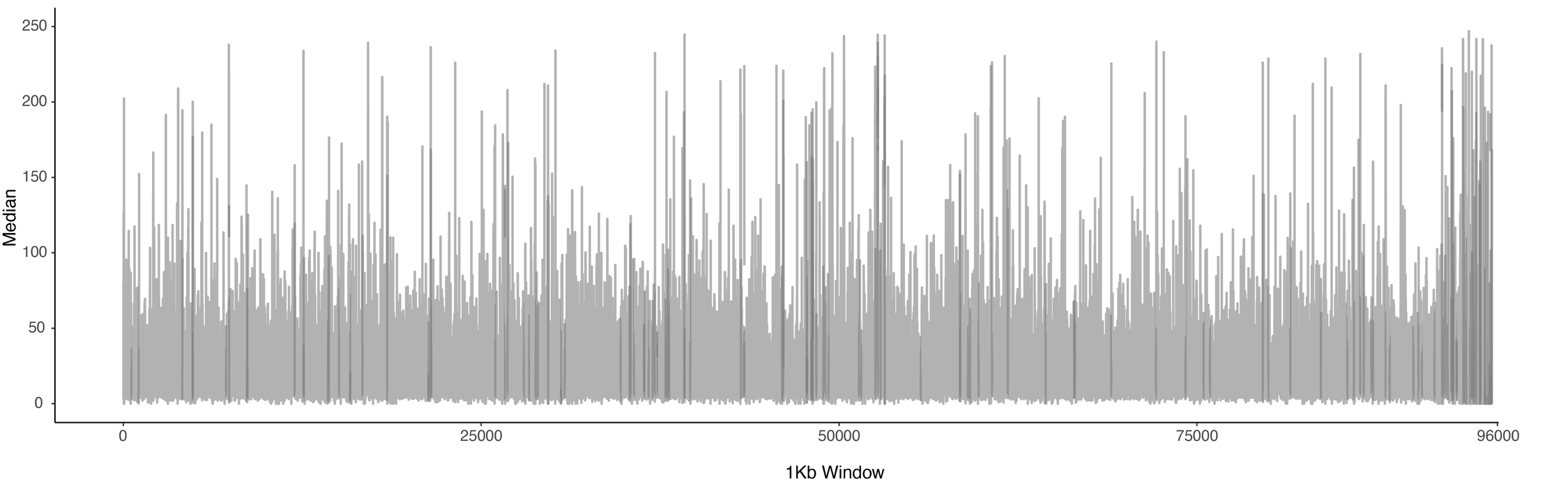

Supplement: S3 Fig — (TIF) [file pntd.0009020.s006.tif]

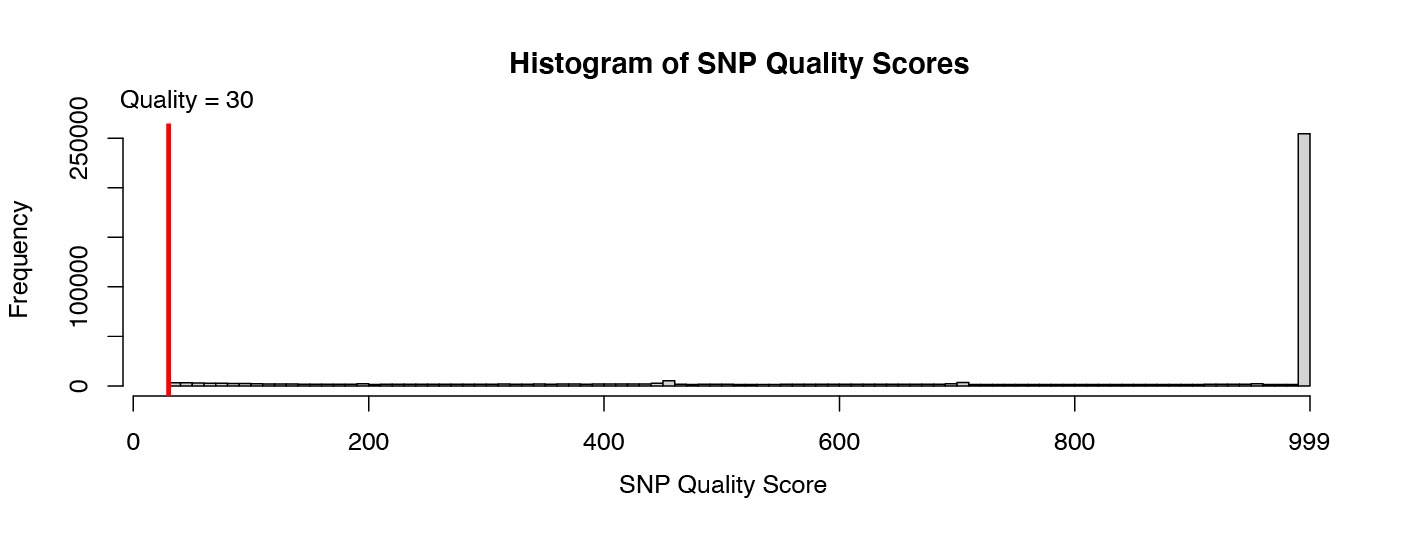

Supplement: S4 Fig — The red bar denotes the minimum quality SNP score of 30. (TIF) [file pntd.0009020.s007.tif]

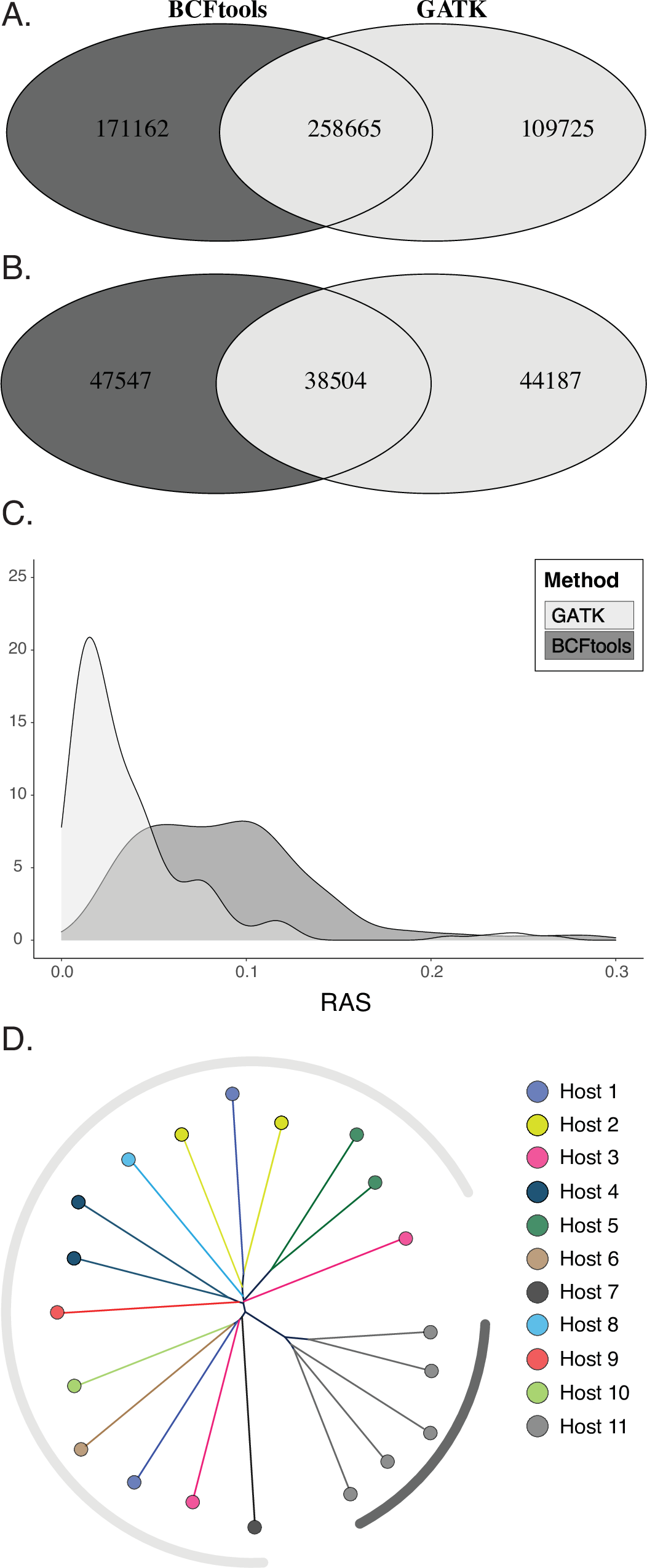

Supplement: S5 Fig — A) Venn diagram showing the number of exonic SNPs that were specific to each variant calling pipeline and the overlap between each method. B) Venn diagram showing the number of rare allele variants specific and shared between each variant calling pipeline. C) Rare allele sharing distributions for each variant calling pipeline. D) Neighbor-joining tree for all exonic variants called using GATK. (TIF) [file pntd.0009020.s008.tif]

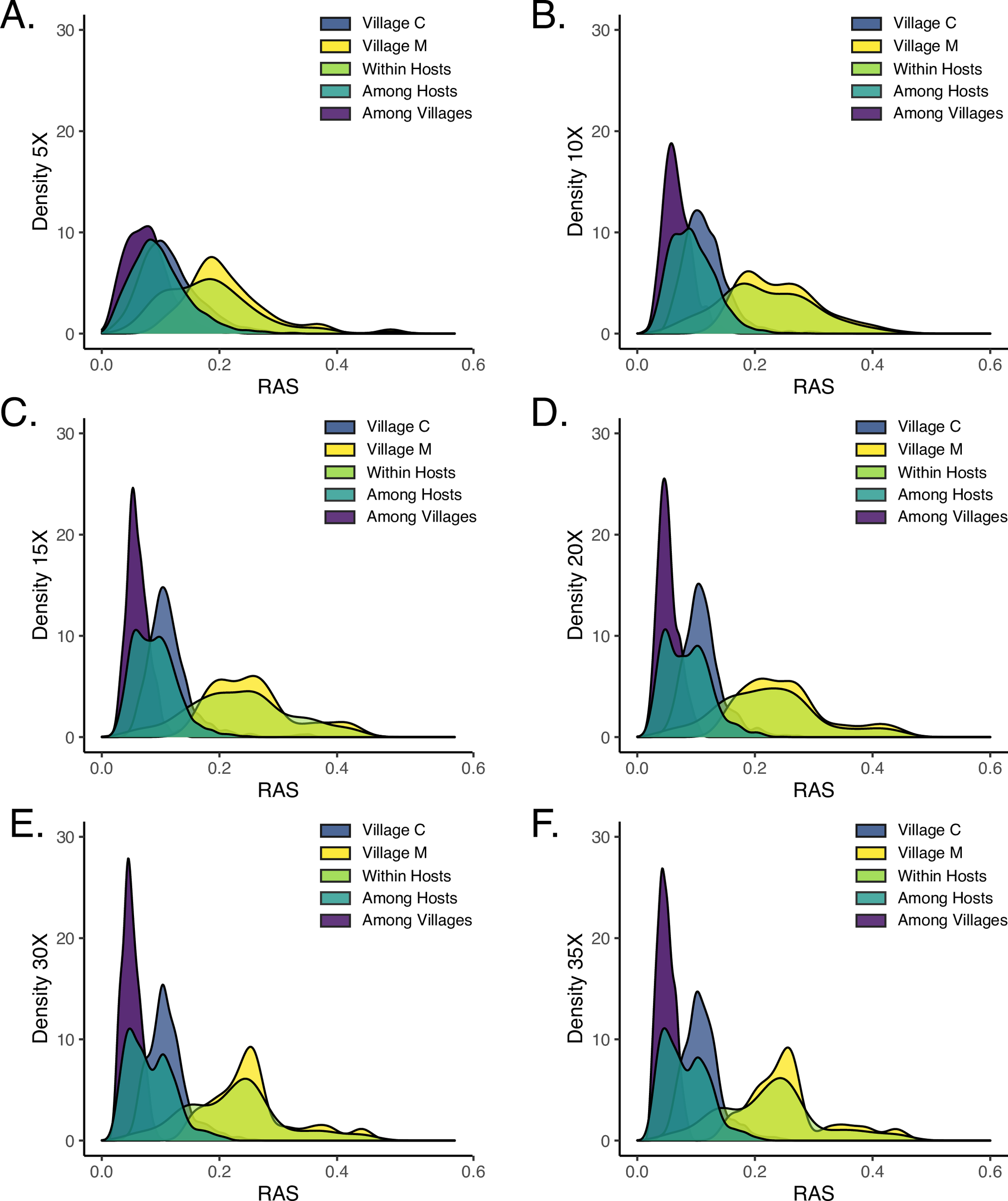

Supplement: S6 Fig — Panels A-F show the distribution of RAS values between pairs of samples from exonic variants for among and between villages/hosts of all downsampled coverage datasets. Colors correspond to the same categories labeled in Fig 2 of the main text. (TIF) [file pntd.0009020.s009.tif]

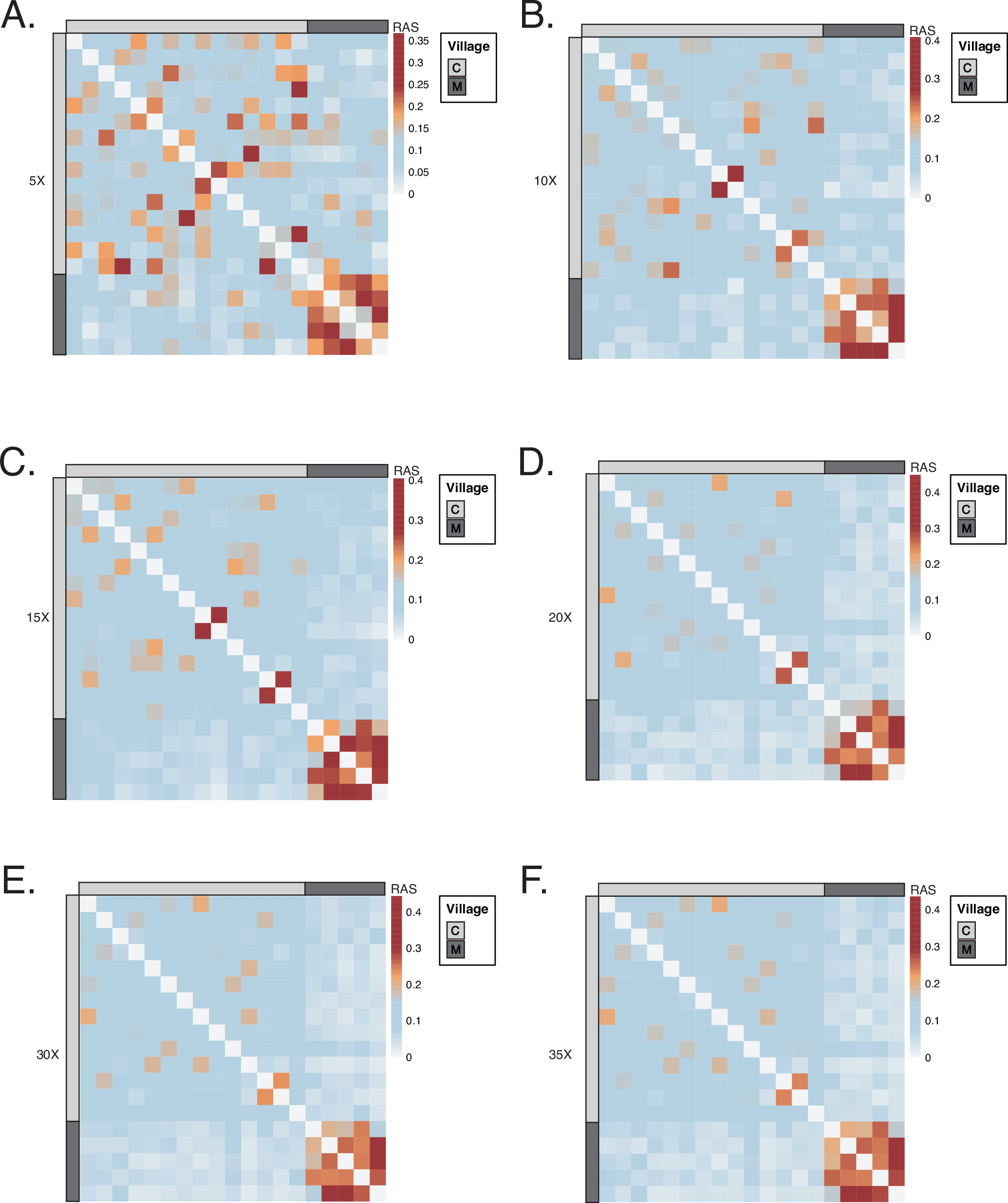

Supplement: S7 Fig — Panels A-F show pairwise comparisons of rare allele sharing for all downsampled coverage datasets with village identity highlighted by the colored bars above. (TIF) [file pntd.0009020.s010.tif]

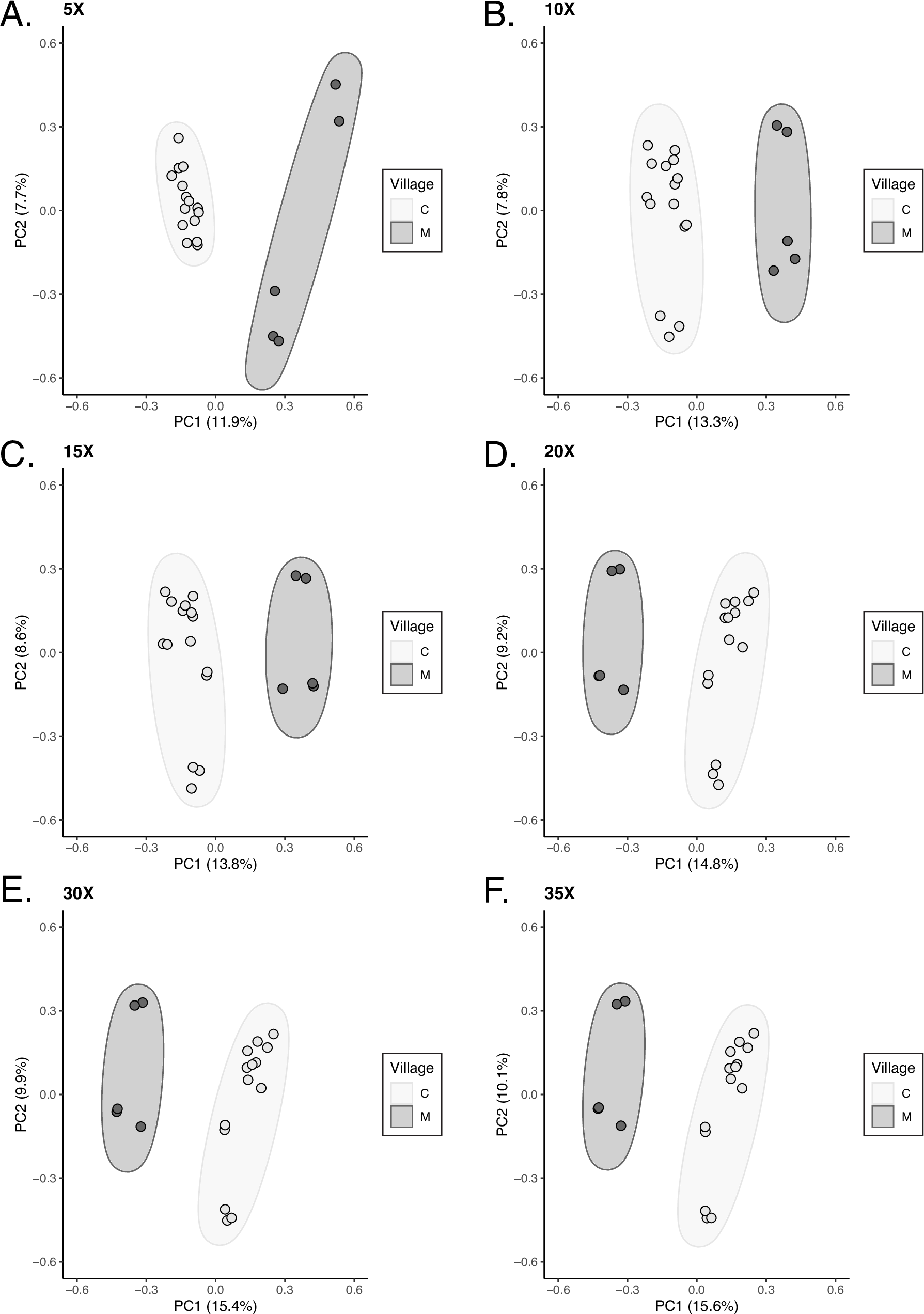

Supplement: S8 Fig — Panels A-F show principal component analysis (PCA) of simulated downsampled coverage. Overall percentage of explained variance are noted next to each PC axis (TIF) [file pntd.0009020.s011.tif]

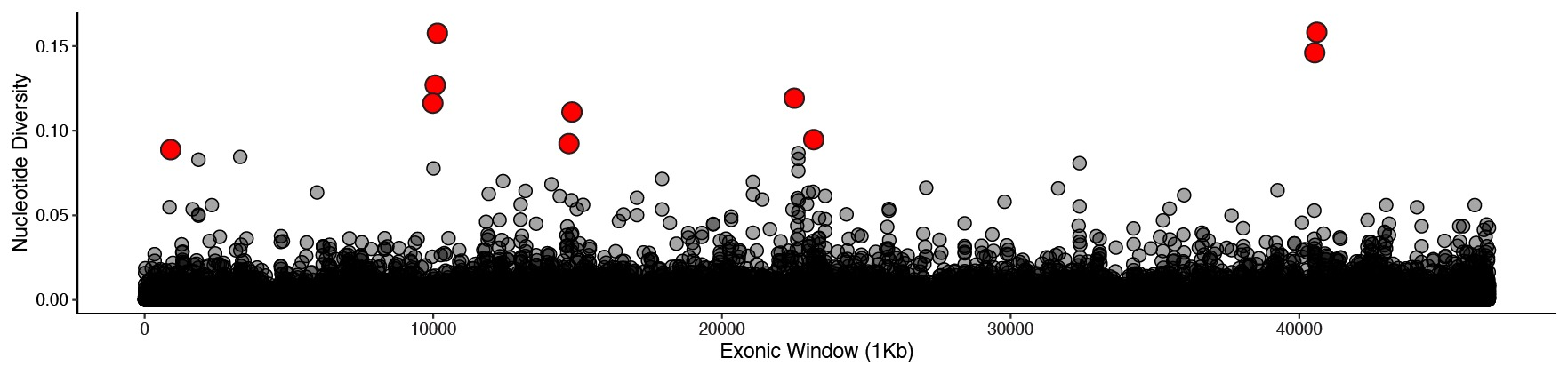

Supplement: S9 Fig — The ten windows with the highest nucleotide diversity are indicated by red dots, and all other values of nucleotide diversity are indicated with gray dots. (TIF) [file pntd.0009020.s012.tif]
